# Ethical issues in assertive outreach and crisis intervention teams

**DOI:** 10.1192/j.eurpsy.2023.401

**Published:** 2023-07-19

**Authors:** J. J. Martínez Jambrina

**Affiliations:** PSYCHIATRY, HOSPITAL SAN AGUSTÍN, AVILES, Spain

## Abstract

**Introduction:**

Assistance to people suffering serious mental illnesses has undergone large variations in the last twocenturies. After World War II, The community became a new destination for them.But hardly anyone knew how to cope with mental illness in thecommunity. Thus, several initiatives arose in the US. Both social work and psychiatry,moved towards a pragmatic point of view by the rising tide of patients seeking help: initiatives trying to providesolutions to themost basic needs of patients: accommodation, food,medical care, takingof medication, etc. By the 1970s, Mary Ann Test and LeonardStein had proven the effectiveness of theirLife Coaching in Madison, Wisconsin. In 1981 his program is disseminated by several states under the name of Assertive TreatmentCommunity and thus spread throughout the United Statesand Canada, Australia and Europe. Psychiatry has recognized it as the program that got the most for supporting the community model. 50 years later the basics of the TAC model remain more or less thesame. But home interventions caused a continuous conflict in the ethical field notwell addressed…

**Objectives:**

WHY IS AN ACT team a fertile ground for ethicalconflicts?This approach is coercive or assertive?There are several reasons. There is a specific ETHICAL ENVIRONMENT in THE ACT team

1.“Diffusion of Responsibility”-

2. Mutual confirmation bias:

3. There is a tendency to think that professionals areethical by nature.

4. Biased search for information when problems arise.

Sources are sought to confirm us before clarifying whathappened

5. A special tendency to conformism.

6.Repetitive responses.

**Methods:**

We will analyze the main ethical conflicts arising in ACT teams:

1. CONFLICTS OF AUTONOMY

2. PRIVACY AND CONFIDENTIALITY ISSUES

3. CONFLICTS OF DUTIES

4. ASSERTIVENESS VERSUS COERCION

**Results:**

The great challenge is knowing how and when to intervenewith patients with variable decision-making capacity orwithout any insight, as well as the impact on theirautonomy. It is an exercise both in art and inphenomenological training: Because there are subtledeficits, difficult to appreciate, but there are otherdeficits that are obvious.

**Conclusions:**

The challenge: balancing the needs and safety of thecommunity with the needs and safety of the individual.ACT teams staff must juggle both perspectives, whilemaintaining a therapeutic alliance.

The continuous contact with the patient in an ACT teamgives, especially to clinicians, a privileged place ofobservation to act correctly in those situations and to be asupport so that whoever arrives lacking in affectivity orwith relational problems could grow until reaching a moreprudent and competent judgment.

WHAT COULD HAPPEN IF WE TRAIN PROFESSIONAL IN BIOETHICS MORE IN PATIENT´S RIGHTS, A FIELD FOR LAWYERS??

**Disclosure of Interest:**

None Declared

